# Evolution of H5N1 Avian Influenza Viruses in Asia

**DOI:** 10.3201/eid1110.050644

**Published:** 2005-10

**Authors:** The World Health Organization Global Influenza Program Surveillance Network

**Keywords:** Influenza, Influenza A Virus, Avian, hemagglutinin, NA protein, M2 protein, molecular Evolution, Phylogeny, virology, Influenza Vaccines, Adamantane, Oseltamivir, research

## Abstract

An outbreak of highly pathogenic avian influenza A (H5N1) has recently spread to poultry in 9 Asian countries. H5N1 infections have caused >52 human deaths in Vietnam, Thailand, and Cambodia from January 2004 to April 2005. Genomic analyses of H5N1 isolates from birds and humans showed 2 distinct clades with a nonoverlapping geographic distribution. All the viral genes were of avian influenza origin, which indicates absence of reassortment with human influenza viruses. All human H5N1 isolates tested belonged to a single clade and were resistant to the adamantane drugs but sensitive to neuraminidase inhibitors. Most H5N1 isolates from humans were antigenically homogeneous and distinct from avian viruses circulating before the end of 2003. Some 2005 isolates showed evidence of antigenic drift. An updated nonpathogenic H5N1 reference virus, lacking the polybasic cleavage site in the hemagglutinin gene, was produced by reverse genetics in anticipation of the possible need to vaccinate humans.

## Abstract 2

Human infections were from a virus clade undergoing antigenic drift that showed resistance to adamantanes but sensitivity to neuraminidase inhibitors.

Highly pathogenic avian influenza viruses of the H5N1 subtype are circulating in eastern Asia with unprecedented epizootic and epidemic effects (*1*). Nine Asian countries reported H5N1 outbreaks in poultry in 2004: Cambodia, China, Indonesia, Japan, Laos, Malaysia, South Korea, Thailand, and Vietnam (*1*). Between 2004 and the first 3 months of 2005, a total of 89 laboratory-confirmed human infections, 52 of which were fatal, were reported to the World Health Organization (WHO) by public health authorities in Vietnam, Thailand, and Cambodia. These records indicate that this outbreak of human H5N1 infections is the largest documented since its emergence in humans in 1997 (*2*). Efficient viral transmission among poultry caused the virus to spread regionally, leading to the loss of >100 million birds from disease and culling. In contrast, human-to-human transmission of the virus is exceptional but has been described, most recently in a family cluster in Thailand (*3*).

The 3 viral envelope proteins of influenza A virus are most medically relevant. The hemagglutinin (HA), neuraminidase (NA), and M2 are essential viral proteins targeted by host antibodies or antiviral drugs such as oseltamivir and rimantadine (*4**–**6*). The HA glycoprotein forms spikes at the surface of virions, mediating attachment to host cell sialoside receptors and subsequent entry by membrane fusion. The NA forms knoblike structures on the surface of virus particles and catalyzes their release from infected cells, allowing virus spread. The M2 is a transmembrane protein that forms an ion channel required for the uncoating process that precedes viral gene expression.

We report on phylogenetic, phenotypic, and antigenic analysis of H5N1 viruses from the 2004–2005 outbreak, focusing on these 3 genes, to address questions relevant to the public health response to the outbreak: 1) What is the genetic diversity of H5N1 viruses involved in human infections? 2) Can the relationship between human and avian H5N1 isolates help explain the source of infection? 3) Do genetic changes correlate with enhanced viral transmissibility in humans? 4) How sensitive are H5N1 isolates to antiviral drugs? 5) What is the antigenic similarity between human H5N1 viruses and current candidate vaccines? and 6) Can candidate vaccine reference stocks be developed in time for an effective public health response?

## Methods

All work involving infectious H5N1 influenza was performed in government-approved biosafety level 3–enhanced containment facilities with experimental protocols in compliance with applicable federal statutes and institutional guidelines. Influenza A (H5N1) viruses isolated in Asia and A/Puerto Rico/8/34 (PR8) (H1N1) were propagated in embryonated chicken eggs or in Madin-Darby canine kidney (MDCK) cells. The African green monkey kidney Vero cell line was from a cell bank certified for human vaccine production.

Viral RNA was extracted by using a commercial lysis solution and resin kit and amplified by reverse transcriptase–polymerase chain reaction with specific oligonucleotide primers. Nucleotide sequencing reactions were performed with a cycle sequencing kit and resolved on an ABI 3100 Genetic Analyzer (Applied Biosystems, Foster City, CA, USA). DNA sequence analysis was performed by using version 10 of the GCG sequence analysis package (*7*), and phylogeny was inferred by using a neighbor-joining tree reconstruction method implemented in the Phylip package (*8*).

Postinfection ferret antisera were prepared as previously described (*9*). Hemagglutination inhibition (HI) testing was performed as previously described with turkey erythrocytes (*10*).

Median inhibitory concentration (IC_50_) values for oseltamivir and zanamivir were determined by using NA-Star substrate and Light Emission Accelerator IITM (Applied Biosystems, Bedford, MA, USA) as previously described (*11*). Biological susceptibility to rimantadine was determined by recording the yield of viral progeny in MDCK cells infected with the H5N1 strains of interest at a multiplicity of >10 median egg infectious doses in the absence or presence of 2 μg/mL rimantadine.

Plasmids with full-length cDNA from the 6 internal genes (PB1, PB2, PA, NP, M, NS) of influenza virus PR8 strain (*12*), flanked by human RNA polymerase I (PolI) promoter and polyadenylation site at the 3´ end and a PolI terminator as well as a PolII promoter at the 5´ end, were generated as described previously (*12**–**14*). The cDNA of N1 NA or H5 HA genes of VN/1203/2004 or VN/1194/2004 (VN/04-like) were inserted into plasmids as described above. The 4 basic amino acid codons from the cleavage site of HA were deleted by overlap extension PCR, as described previously (sequences available upon request) (*13**,**15**–**17*).

PR8 reassortant viruses with HA and NA from VN/04-like viruses were generated by plasmid DNA-based reverse genetics in Vero cell under good laboratory practice conditions appropriate for future human use. Candidate vaccine reference reagent reassortant viruses were generated at the National Institute of Biological Standards and Control (NIBSC), South Mimms, United Kingdom; Saint Jude Children's Research Hospital (SJCRH), Memphis, Tennessee, USA; and Centers for Disease Control and Prevention (CDC), Atlanta, Georgia, USA. For brevity, the reverse genetics derivation method described represents a consensus of the institutions; minor unpublished protocol details unique to each laboratory were not described and are available upon request. The VN/04x/PR8 reassortant virus was recovered in embryonated eggs and identified in the allantoic fluid by HA assay. The genetic and antigenic properties of the resulting reassortant virus were determined as described previously (*15**,**18**–**20*). Candidate vaccine stocks were subjected to virulence studies in avian, murine, and ferret models to establish their safety (*19*).

## Results

### Analysis of HA, NA, and M2 Genes from H5N1 Viruses

**Figure 1:**
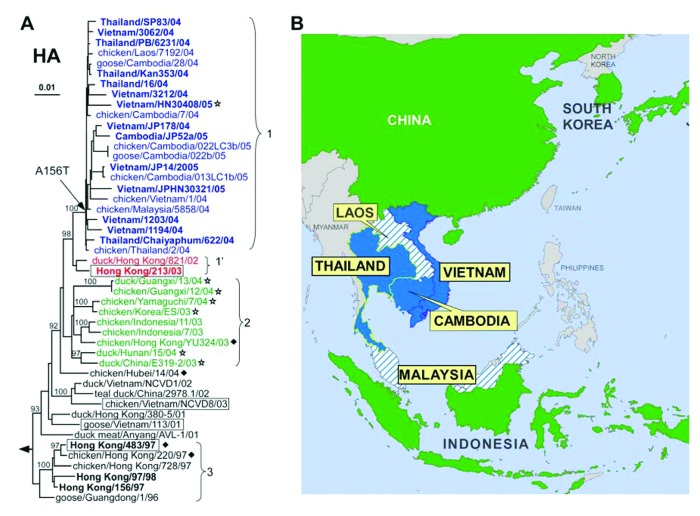
Phylogenetic relationships among H5 hemagglutinin (HA) genes from H5N1 avian influenza viruses and their geographic distribution. Viral isolates collected before and during the 2004–2005 outbreak in Asia and selected ancestors were included in the analysis (Table A1). HA clades 1, 1′, and 2, discussed in the text, are colored in blue, red, and green fonts, respectively. Virus names in **boldface** denote isolates from human infections. Phylogenetic trees were inferred from nucleotide sequences by the neighbor-joining method with A/chicken/Scotland/56 genes as outgroup (not shown, denoted by arrowhead). Bootstrap analysis values >90% are shown. A) HA gene tree phylogeny was based on the coding region of the segment. Presence of a motif for glycosylation in HA is indicated as A156T by an arrow at the root of clade 1 and a diamond for other clades (Table 1). Stars denote absence of 1 arginine residue at the polybasic cleavage site, which starts at position 325 of HA1. Isolates to which ferret antisera were made for antigenic analyses are boxed (Table 2). B) Geographic distribution of H5N1 in east Asia: blue denotes countries reporting infections with clade 1 H5N1 in humans and birds (solid) or in birds only (hatched). Green denotes countries reporting bird infections with clade 2 H5N1 viruses.

Phylogenetic analyses of the H5 HA genes from the 2004 and 2005 outbreak showed 2 different lineages of HA genes, termed clades 1 and 2. Viruses in each of these clades are distributed in nonoverlapping geographic regions of Asia (Figure 1). The H5N1 viruses from the Indochina peninsula are tightly clustered within clade 1, whereas H5N1 isolates from several surrounding countries are distinct from clade 1 isolates and belong in the more divergent clade 2. Clade 1 H5N1 viruses were isolated from humans and birds in Vietnam, Thailand, and Cambodia but only from birds in Laos and Malaysia. The clade 2 viruses were found in viruses isolated exclusively from birds in China, Indonesia, Japan, and South Korea. Viruses isolated from birds and humans in Hong Kong in 2003 and 1997 made up clades 1´ and 3, respectively.

The HA genes from H5N1 viruses isolated from human specimens were closely related to HA genes from H5N1 viruses of avian origin; human HA gene sequences differ from the nearest gene from avian isolates from the same year in 2–14 nucleotides (<1% divergence). These findings are consistent with the epidemiologic data that suggest that humans acquired their infections by direct or indirect contact with poultry or poultry products (*21*).

Analysis of the amino acid sequences showed that both clades of H5 HAs from the 2004–2005 outbreak have a multiple basic amino acid motif at the cleavage site, a defining feature of highly pathogenic avian influenza viruses. Among all H5N1 isolates collected in east Asia since 1997, only those in clades 1, 1´, and 3 appear to be associated with fatal human infections (*22**,**23*). We compared amino acid sequences of HA from contemporary isolates (clades 1 and 2) with those of the fatal H5N1 infections in Hong Kong in 1997 and 2003 to identify changes that may correlate with patterns of human infection (Table 1). Thirteen polymorphic sites were identified when the HA1 from the 4 consensus sequences were compared. One change in the 2004–2005 viruses is serine 129 to leucine (S129L). This change affects receptor binding because S129 makes atomic contact with cellular sialoside receptors (*24*). A second structural change in HA was the A156T substitution, which resulted in glycosylation of asparagine 154 and is predicted to reduce its affinity for sialosides. This change is commonly associated with viral adaptation to terrestrial poultry and increased virulence for these birds (*25**–**27*).

**Table 1:** Amino acid differences among H5 hemagglutinins (HA1)

Clade 3	Clade 2	Clade 1´	Clade 1	H3 No.	Functional significance
N45*	D	D	D	54†	Antigenic site C
S84	N	N	N	92	Antigenic site E
A86	A	A	V	93	Antigenic site E
N94	D	D	D (1)	101	Near Y91; receptor binding?
N124	D	S	S	129	Antigenic site B
S129	S	L	L	133a	Receptor binding
L138	Q	Q	Q	142	Antigenic site A
S155	–	N155	–	159	Antigenic site B
T156‡	A	A	T	160	N154 glycosylation motif
L175	L	L	L (2)	179	Near H179; receptor binding?
T188	T	T	T (3)	192	Near L190; receptor binding?
K189	R	R	K	193	Adjacent to receptor binding, antigenic site B
E212	K	K	R	216	Antigenic site D
S223	–	N223§	– (4)	227	Receptor binding
T263	A	A	T	266	Antigenic site E
325R¶	Absent	–	–	Absent	HA cleavage efficiency

*Amino acid residue in single-letter code and position in the mature H5 HA1. †Equivalent residue number in the mature H3 HA1 aligned with H5 amino acid sequence; –, no change from HK97 clade HA consensus. ‡A156 or S156 were found in certain clade 3 HAs; A156 was present in some HAs from clade 2. HA genes from »50% of isolates collected in 2005 had these substitutions present in only one isolate: 1) to N or V; 2) to M or I; 3) to A, V, or I; or 4) to N. §Change present exclusively in isolates from humans. ¶Arginine at the start of the polybasic cleavage site, position 325.

Because of the heightened alert due to H5N1 infections in Vietnam during the first months of 2005, we examined the HA sequences for evidence of shared amino acid changes. The HA of viruses isolated in the first 3 months of 2005 showed several amino acid changes relative to 2004 viruses (Table 1). None of the changes in the HA were common to all the 2005 viruses, which suggests that these variant viruses are cocirculating independently in poultry. The most commonly observed changes are located within short distances of the receptor-binding site. For example, positions D94, L175, and T188 may modulate the interaction of Y91, H179, and L190 with sialosides. One of the isolates from a fatal infection in 2005 showed a substitution of serine 223 to asparagine, which is predicted to facilitate binding of sialosides commonly found in mammalian species (Table 1).

**Figure 2:**
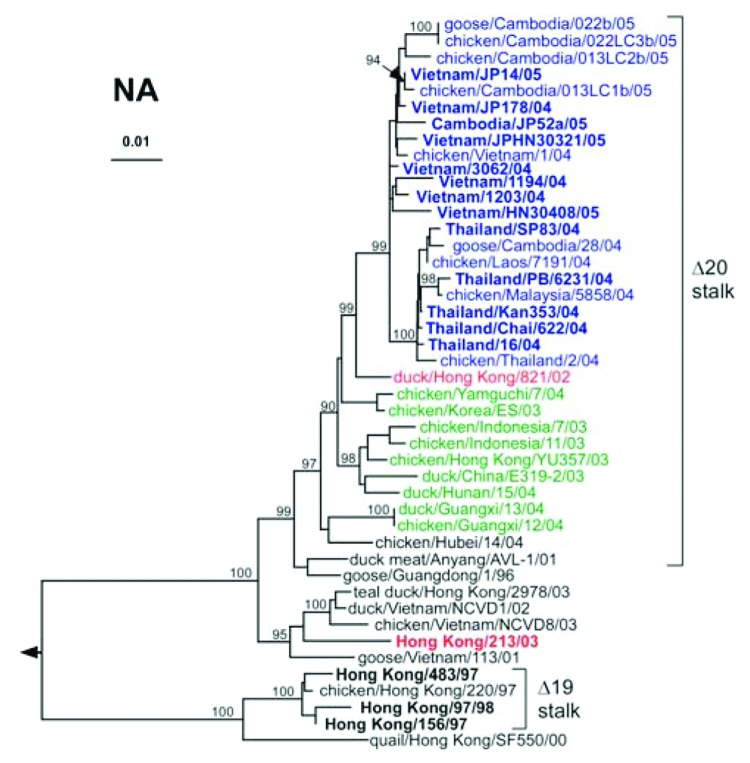
Phylogenetic relationships among N1 neuraminidase (NA) genes of H5N1 influenza viruses. The clade of the hemagglutinin of each of these viruses is indicated by font coloring as in Figure 1A. Brackets denote genes encoding NA protein with deletions in the stalk region; residues 49–68 for clades 1–2 and 57–75 in clade 3.

The phylogenetic tree of the NA genes resembled that of the HA genes, which indicates coevolution of these 2 envelope genes (Figure 2). NA genes of isolates from Thailand seem to have diverged to form a group distinct from that of genes from Vietnam viruses. As reported previously, the NA of HK/213/03 did not co-evolve with the HA genes (*28*). NA genes from human and related avian H5N1 isolates from 2003–2005 as well as clade 3 isolates were characterized by deletions in the stalk region of the protein (positions 49–68 for clades 1–2 and 54–72 for clade 3) (*29*). Deletions in the stalk of the NA are thought to increase retention of virions at the plasma membrane (*30*) to balance weaker binding of sialic acid receptors by the HA with newly acquired N154 glycosylation.

Neuraminidase inhibitors are effective antiviral drugs against human influenza viruses, and preclinical studies suggest a similar effectiveness against avian influenza in humans (*5**,**31*). The IC_50_ of oseltamivir for the clade 1 and 2 NA of 2004–2005 isolates was <10 nmol/L, as compared to IC_50_ values of 85 and 1,600 nmol/L for resistant H1N1 or H3N2 mutants used as controls (Table 3). Thus, NA of H5N1 isolates is sensitive to this class of antiviral agents.

**Table 3:** Sensitivity of H5N1 influenza isolates to oseltamivir

Virus	Oseltamivir IC_50_*
H1N1 (H274)†	0.69
H1N1 (Y274)†	85.92
H3N2 (R292)‡	1.99
H3N2 (K292)‡	1,600.00
Hong Kong/483/97	4.86
Hong Kong/213/03	5.07
Vietnam /1194/04	2.49
Vietnam/1203/04	7.68
Chicken/VN/NCVD1/04	5.87
Chicken/VN/NCVD8/03	9.90

*Median inhibitory concentration (IC_50_) of oseltamivir (nmol/L) for H5N1 influenza isolates and control H1N1 or H3N2 isolates (results for viruses shown are representatives of 31 isolates tested). †Wildtype (H274) and resistant mutant (Y274) influenza virus A/Texas/36/91 (H1N1). ‡Wildtype (R292) and resistant mutant (K292) influenza virus A/Victoria/3/75 (H3N2).

The phylogenetic tree of the M genes resembled that of the HA genes, indicating coevolution of these genes (results not shown). The amino acid sequence of the M2 protein of clade 1 viruses as well as of HK/213/03 indicated a serine-to-asparagine substitution at residue 31 (S31N), known to confer resistance to adamantanes (including amantadine and rimantadine) (*6*). Clade 1 isolates from 2004 and 2005 cultured in the presence of 2 μg/mL rimantadine replicated as efficiently as in untreated cultures, whereas the replication of HK/483/97 was reduced to 1% of control values, indicating that all the currently circulating clade 1 isolates are resistant to adamantanes (data not shown).

### Origin of Internal Genes of H5N1 Viruses from Asia

A complete genetic characterization of circulating H5N1 viruses is critical to identify the possible incorporation of human influenza virus genes by reassortment. To this end, we analyzed the phylogeny of the internal protein coding genes. The PB2, PB1, and PA polymerase genes from 2003–2005 H5N1 isolates from humans constitute a single clade (data not shown) and have coevolved with the respective HA genes (Figure 1). No evidence of reassortment with polymerase genes from circulating H1N1 or H3N2 human influenza virus was found. The phylogenies of the NP and NS genes also supported the avian origin of these genes, indicating that all the genes from the human H5N1 isolates analyzed are of avian origin, which confirms the absence of reassortment with human influenza genes. Taken together, the phylogenies of the 8 genomic segments show that the H5N1 viruses from human infections and the closely related avian viruses isolated in 2004 and 2005 belong to a single genotype, often referred to as genotype Z (*1*).

### Antigenic Analysis of H5N1 Viruses from Asia

Influenza vaccines whose HA are antigenically similar to circulating strains provide the highest level of protection from infection (*32*). H5N1 isolates collected in 2004 and 2005 analyzed by the HI test showed reactivity patterns that correlated with the 3 main clades of recent isolates identified in the HA gene phylogeny (Table 3 and Figure 1). Viruses from humans and birds in clade 1, represented by VN/1203/04, were found to constitute a relatively homogeneous and distinct antigenic group characterized by poor inhibition by ferret antisera to isolates from other clades (Table 2), in particular by the ferret antiserum raised to HK/213/03 (64-fold reduction compared to the homologous titer). The latter isolate was previously used to develop a vaccine reference strain in response to 2 confirmed H5N1 human infections in February 2003 (*15*). These HI results provided the motivation for the development of an updated H5N1 vaccine that would be antigenically similar to 2004–2005 human isolates. The antigenic similarity of VN/1203/04 and the closely related VN/1194/04 to the contemporaneous H5N1 isolates from humans (data not shown) prompted their selection for vaccine reference stock development.

**Table 2:** Antigenic analysis of H5N1 isolates from Asia

Virus antigen	Clade	Reference ferret antisera*								
		HK156	NCVD8	HK213	VN1203	VN04xPR8-rg	VN78	VN4207	VN14	VN32321
A/Hong Kong/156/97	3	**1,280**	320	640	80	320	40	80	80	80
A/ck/Vietnam/NCVD8/03	2	640	**160**	80	80	160	20	<10	160	40
A/Hong Kong/213/03	1´	1,280	1,280	**2,560**	80	640	160	160	640	640
A/Vietnam/1203/04	1	40	20	<10	**640**	320	40	160	80	40
A/Vietnam/1203/04xPR8-rg	1	80	<10	10	640	**320**	40	160	160	40
A/Vietnam/1194/04	1	40	20	10	640	320	40	160	160	40
A/Vietnam/JP178/04	1	80	10	<10	1,280	320	**80**	160	160	80
A/Vietnam/JP4207/05	1	160	40	40	1,280	640	80	**320**	160	80
A/Vietnam/JP14/05	1	20	<10	10	640	80	20	40	**80**	40
A/Vietnam/JP30321/05	1	40	40	10	<10	40	10	<10	40	**160**

*Homologous HI titers are in **boldface**.

Antigenic analysis of human isolates from 2005 provided evidence of antigenic drift among the most recently circulating H5N1 strains (Table 2). For example, VN/JPHN30321/05 showed a reduced HI titer against VN/1203/04 reference serum. This antigenic difference is correlated with 7 amino acid differences between the HA1 domain VN/1203/04 and VN/JPHN30321/05: R53K, N84D, D94N, K140R, L175M, K189R, and V219I (Table 1 and Table A1). Development of Candidate H5N1 Vaccine Reference Stocks Mass vaccination is the most effective approach to reduce illness and death from pandemic influenza. Inactivated influenza vaccines are manufactured from reassortant viruses obtained by transferring the HA and NA genes with the desired antigenic properties into a high-growth strain such as PR8 (*33*). However, reassortants with H5-derived HA with a polybasic cleavage site are potentially hazardous for animal health. Because the high pathogenicity of the H5N1 viruses in poultry, mice, and ferrets depends primarily on the polybasic cleavage site in the HA molecule, a derivative with a deletion of this motif was engineered in cloned HA cDNAs. Three high-growth reassortant influenza viruses were developed: NIBRG-14 (NIBSC), VN/04xPR8-rg (SJCRH), and VNH5N1-PR8/CDC-rg (CDC). These candidate vaccine strains, bearing mutant H5 HA, intact NA, and the internal genes from PR8, were generated by a reverse genetics approach (*12**,**13**,**20**,**34*) using Vero cells and laboratory protocols compatible with eventual use of the vaccine in human subjects (*15**,**18*). These 3 vaccine candidates were characterized genetically (nucleotide sequencing of HA and NA) and antigenically in HI assays to confirm that their antigenicity remained unchanged relative to the wildtype virus (Table 2). The candidate reference stocks had molecular and antigenic properties equivalent to parental H5N1 donor strains and lacked virulence in chicken, mouse, and ferret models.

## Discussion

The growing H5N1 epizootic in eastern Asia could expand the environmental load of virus and cause more infections in mammals (*35*), which would increase the probability that a highly transmissible virus will emerge in mammals. We therefore analyzed the medically relevant genes from viruses isolated from the beginning of the outbreak until March 2005 to evaluate parameters relevant to public health.

The origin of the HA genes of the 2004–2005 outbreak as well as an earlier isolate from a fatal human infection in Hong Kong in 2003 (clade 1´) can be traced back to viruses isolated in 1997 in Hong Kong (clade 3) and from geese in China (goose/Guangdong/96) (Figure 1A). The phylogeny also shows that viruses with HK/97-like HA may have circulated in avian hosts continuously after 1997, without causing any reported human infections until the 2 confirmed cases in Hong Kong in February 2003 (*28*).

The 2004–2005 H5N1 isolates are sensitive to 2 neuraminidase inhibitors that are recommended for prophylactic or therapeutic intervention against human infections with recent H5N1 strains. Rapidly testing potentially pandemic influenza viruses for their susceptibility to licensed drugs is essential to establish appropriate control measures.

An effective H5N1 vaccine is a public health priority and the cornerstone for pandemic prevention and control. Reverse genetics approaches allow the rapid production of high-growth PR8 reassortant viruses by engineering a virus with a homologous HA gene lacking the polybasic amino acids associated with high virulence. These candidate H5N1 pandemic vaccine viruses have been made available to vaccine manufacturers to produce pilot lots for clinical trials and are available for possible large-scale manufacturing should the need arise.

Genetic and antigenic analyses have shown that, compared to previous H5N1 isolates, 2004–2005 isolates share several amino acid changes that modulate antigenicity and perhaps other biological functions. Furthermore, our molecular analysis of the HA from isolates collected in 2005 suggests that several amino acids located near the receptor-binding site are undergoing change, some of which may affect antigenicity or transmissibility. For example, an isolate (VN/JP12-2/05) showed a change from serine to asparagine at position 223 of the HA1 (S223N) that may affect receptor-binding specificity (*36*). The VN/30321/05 isolate demonstrated considerable antigenic drift from VN/04-like isolates, which have been selected as the candidate vaccine antigens. Further surveillance to determine the prevalence of such variants in poultry will be critical to determine if these variants compromise the efficacy of the candidate vaccine or increase the efficiency of transmission.

The phylogenies of the 8 genomic segments from the clade 1 and 2 isolates from 2004–2005 showed that all genes are of avian origin. All H5N1 isolates from both clades belong to 1 of the genotypes recently circulating in Eastern and Southern Asia, e.g., genotypes V and Z (*1**,**37*). The influenza virus genome has remarkable plasticity because of a high mutation rate and its segmentation into 8 separate RNA molecules. This segmentation allows frequent genetic exchange by segment reassortment in hosts co-infected with 2 different influenza viruses. No evidence has been seen that the 2004–2005 H5N1 isolates have acquired nonavian influenza genes by reassortment. However, continued surveillance is important because genetic reassortment may facilitate the evolution of viruses with increased virulence or expanded host range.

The currently circulating H5N1 viruses were reported to infect domestic or wild captive felids, such as tigers, feeding on infected bird carcasses, and the infected cats can transmit H5N1 to pen mates (*38*). Furthermore, circumstantial evidence indicates that tiger-to-tiger transmission of H5N1 has occurred at a zoo in Thailand (*39*). Recent evidence of person-to-person transmission and the clustering of H5N1 cases raise the level of concern for a pandemic of H5N1 influenza (*3*). Therefore, sustained and aggressive efforts to control H5N1 circulation in poultry are mandatory to avoid possible catastrophic public health consequences.

## Appendix

**Table A1:** Influenza Sequence Database accession numbers of influenza virus genes analyzed*

Influenza strain	Clade	Hemagglutinin	Neuraminidase	M
A/Vietnam/HN30408/05	1	ISDN119678	ISDN119679	NA†
**A/Vietnam/JP14/05**	**1**	**ISDN117778**	**ISDN117783**	**NA**
**A/Vietnam/JPHN30321/05**	**1**	**ISDN118371**	**NA**	**NA**
**A/Vietnam/JP4207/05**	**1**	**ISDN117777**	**ISDN117782**	**NA**
A/Cambodia/JP52a/2005	1	ISDN121986	ISDN122818	NA
A/Chicken/Cambodia/013LC1b/2005	1	ISDN122143	ISDN122148	NA
A/Chicken/Cambodia/013LC2b/2005	1	NA	ISDN122151	NA
A/Chicken/Cambodia/022LC3b/2005	1	ISDN122146	ISDN122152	NA
A/Goose/Cambodia/022b/2005	1	ISDN122147	ISDN122153	NA
**A/Vietnam/JP178/04**	**1**	**ISDN69608**	**ISDN69610**	**AF255363**
A/Vietnam/1194/2004	1	ISDN38686	ISDN38703	ISDN39957
**A/Vietnam/1203/2004**	**1**	**ISDN38687**	**ISDN38704**	**ISDN39958**
A/Thailand/16/2004	1	ISDN40341	ISDN48790	ISDN45755
A/Vietnam/3062/2004	1	AY651336	AY651448	NA
A/Vietnam/3212/2004	1	ISDN40278	NA	NA
A/Thailand/Suphanburi/83/2004	1	ISDN40917	ISDN48792	ISDN111182
A/Thailand/Kan353/2004	1	ISDN40918	ISDN48791	1SDN111183
A/Thailand/Chaiyaphum/622/2004	1	ISDN49460	ISDN48793	ISDN111184
A/Chicken/Thailand/2/2004	1	ISDN49021	ISDN49267	ISDN49264
A/Prachinburi/6231/2004	1	ISDN110940	ISDN110939	ISDN111185
A/Chicken/Malaysia/5854/2004	1	ISDN80767	ISDN80771	ISDN80770
A/Chicken/Vietnam/1/2004	1	ISDN40909	ISDN48796	ISDN111226
A/Duck/Vietnam/NCVD1/2002	–	ISDN38689	ISDN38697	ISDN39960
**A/Chicken/Vietnam/NCVD8/2003**	**–**	**ISDN40326**	**ISDN38706**	**ISDN39967**
A/Chicken/Laos/7191/2004	1	ISDN40922	ISDN48817	ISDN111227
A/Chicken/Laos/7192/2004	1	ISDN40923	NA	NA
A/Goose/Cambodia/28/2004	1	ISDN49121	ISDN49130	ISDN49144
A/Chicken/ Cambodia/7/2004	1	ISDN49117	NA	NA
A/Duck/Hong Kong/821/2002	1´	AY575874	ISDN38794	AY575898
**A/Hong Kong/213/2003**	**1´**	**ISDN38262**	**ISDN48789**	**AY575894**
A/Chicken/Guangxi/12/2004	2	ISDN48980	ISDN48982	ISDN48983
A/Duck/Guangxi/13/2004	2	ISDN48989	ISDN48987	ISDN48986
A/Duck/Hunan/15/2004	2	ISDN48957	ISDN48958	ISDN48968
A/Chicken/Indonesia/7/2003	2	ISDN111351	ISDN111353	ISDN111355
A/Chicken/Indonesia/11/2003	2	ISDN111352	ISDN111354	ISDN111356
A/Chicken/Yamaguchi/7/2004	2	ISDN49016	ISDN49017	ISDN49085
A/Chicken/Korea/ES/2003	2	ISDN40921	ISDN38694	ISDN45748
A/Chicken/Hong Kong/YU324/03	2	AY651358	NA	NA
A/Chicken/Hong Kong/YU35703	2	NA	ISDN38805	ISDN38768
A/Duck/China/E319-2/2003	2	AY518362	AY518363	AY518361
A/Chicken/Hubei/14/2004	–	ISDN48972	ISDN48973	ISDN48975
A/ Teal/Hong Kong/2978/2002	–	AY651352	ISDN38789	AY651417
A/Goose/Vietnam/113/2001	–	ISDN38260	ISDN48794	ISDN117743
A/Duck/Hong Kong/380.5/2001	–	AY075033	AY075034	AY075035
A/Duck meat/Anyang/AVL-1/2001	–	AF468837	AF468838	AF468843
A/Quail/Hong Kong/SF550/2000	3	NA	AJ410561	AJ410571
**A/Hong Kong/156/97**	**3**	**AF460880**	**AF036357**	**AF036358**
A/Hong Kong/483/97	3	AF460970	AF102668	AF255367
A/Hong Kong/97/98	3	AF102676	AF102661	AF255374
A/Aquatic bird/Hong Kong/M603/98	NA	NA	AF098551	AF250486
A/Chicken/Hong Kong/728/97	3	AF046099	NA	NA
A/Chicken/220/97	3	AF046080	NA	NA
A/Goose/Guangdong/1/96	3	AF144305	AF144304	AF144306
A/Hong Kong/1073/99	NA	NA	NA	Submit

*Virus names shown in **boldface** were used to prepare antiserum for use in antigenic characterization (Table 3). NA, not applicable. †ISDN numbers are found in the Los Alamos National Laboratory Influenza Sequence Database (available at http://apollo.lanl.gov); others are in Genbank (http://www.ncbi.nlm.nih.gov/entrez/query.fcgi?db=Nucleotide).
